# Beverage-Induced Staining and Water Sorption/Solubility of Conventional and Resin-Modified Glass-Ionomer Restoratives

**DOI:** 10.3390/biomimetics11040249

**Published:** 2026-04-04

**Authors:** Fatin A. Hasanain, Rotana M. Abulaban, Nouf S. Almeganni, Hani M. Nassar

**Affiliations:** Department of Restorative Dentistry, Faculty of Dentistry, King Abdulaziz University, Jeddah 21589, Saudi Arabia

**Keywords:** color stability, glass ionomer cements, restorative dentistry, solubility, water sorption

## Abstract

Glass ionomer cements (GICs) are considered functionally biomimetic as they participate in ion-exchange processes that partially resemble the behavior of natural enamel and dentin, chemically bond to dental hard tissues, and release fluoride. While GICs are designed to interact with aqueous oral environments, their exposure to dietary beverages may affect their esthetic stability and water-related behavior within the oral environment. For biomimetic restorative materials to perform successfully in the oral environment, they must maintain not only bioactive properties but also esthetic stability and resistance to water-related degradation during exposure to dietary beverages. This study evaluated beverage-induced color changes, water sorption, and water solubility of six GICs following their immersion in coffee, tea, berry juice, cola, and distilled water (*n* = 5 per material per solution). Color measurements were recorded at baseline and after 2, 4, 6, and 8 weeks using a spectrophotometer, and color change (ΔE) values were calculated using the CIE L*a*b* system. Specimen mass was measured at baseline, after 8 weeks of immersion and then after 4 weeks of desiccation. Data were analyzed using repeated-measures Analysis of Variance (ANOVA) and Fisher’s least significant difference post hoc tests (α = 0.05). The results showed time, material, and solution significantly affected ΔE (*p* < 0.001). Tea produced the greatest discoloration overall, followed by coffee. ChemFil exhibited the greatest staining susceptibility, while Fuji II showed the lowest staining susceptibility. Water sorption and solubility were material- and solution-dependent. Clinically relevant discoloration of GICs was found when immersed in common beverages over time, with tea showing the strongest staining effect. These findings indicate that although GICs exhibit biomimetic characteristics through their interaction with tooth structures and aqueous environments, their long-term esthetic stability and resistance to environmental challenges should also be considered when selecting restorative materials for clinically visible areas.

## 1. Introduction

The aim of biomimetic dentistry is to restore tooth structure using materials that replicate the functional, physical and chemical behavior of natural teeth along with their appearance. In the myriad of available restorative materials, glass ionomer cements (GICs) are considered functionally biomimetic. This is due to their ability to chemically bond to dental hard tissue, release fluoride and participate in ion exchange processes that resemble some aspects of the behavior of dentin and enamel [1].

The biomimetic concept goes beyond chemical bonding and fluoride release; it also encompasses the ability of restorative materials to interact dynamically with the aqueous oral environment [1]. GICs rely on water-mediated ion exchange processes that support their bioactive behavior and maturation [1,2]. Interestingly, these same interactions may also influence their susceptibility to discoloration and structural changes when exposed to dietary beverages. Therefore, evaluating how these materials respond to aqueous and chromogenic environments is important for understanding how well they maintain both functional and esthetic integration with natural tooth structures over time.

Conventional GICs and resin-modified glass ionomer cements (RMGICs) were developed to balance bioactivity with improved mechanical and handling properties. While RMGICs were introduced to overcome limitations such as moisture sensitivity and low early strength, their resin components may alter water interaction behavior and long-term esthetic performance [1,2]. Because biomimetic restorative materials are expected to maintain both functional and esthetic integration with surrounding tooth structures, resistance to discoloration and degradation in the oral environment is clinically significant.

A major esthetic failure of restorative materials is color change, especially due to food and beverage consumption [3]. The staining potential and color stability of conventional GICs have been previously investigated by several researchers [4,5,6]. The consumption of beverages such as tea, coffee and soft drinks is not only associated with color changes in the restorative materials, they may also affect the quality of these restorations [7]. The color stability of RMGICs has not received as much attention from the research community [8]. Previous work has shown that the stainability of biomaterials is affected by their water sorption characteristics [9]. Water plays an important role in the ion-exchange processes of glass-ionomer cements, which is central to their biomimetic behavior. However, prolonged exposure to acidic and aqueous dietary environments may simultaneously support functional adaptation through hydration while promoting ion leaching, surface degradation, and optical instability. Assessing color stability in conjunction with water sorption and solubility allows a more comprehensive evaluation of how these biomimetic materials balance their bioactivity with long-term esthetic integration in the oral environment. However, it is important to note that biomimicry in restorative dentistry is not evaluated solely in terms of bonding ability or fluoride release. Discoloration, water sorption, and solubility may compromise the esthetic integration and durability of restorations over time. Therefore, evaluating color stability together with water sorption and solubility provides a broader understanding of how these biomimetic materials interact with the oral environment during prolonged exposure.

Thus, the current study aims to evaluate the beverage-induced color change, water sorption, and water solubility of conventional and resin-modified glass-ionomer restorative materials after immersion in commonly consumed beverages.

The null hypothesis of this study was that beverage type, immersion time, and material composition would have no significant effect on the color stability, water sorption, or water solubility of the tested GICs.

## 2. Materials and Methods

Six types of GICs were included in this study: Riva light cure HV (SDI, Bayswater, Victoria, Australia), Riva self cure HV (SDI, Bayswater, Victoria, Australia), Fuji II (GC, Tokyo, Japan), Fuji IX (GC, Tokyo, Japan), ChemFil^®^ Rock (Dentsply Sirona, New York, NY, USA), and 3M self cure (SC) Ketac™ Fil Plus Aplicap™ (3M ESPE, Minnesota Mining, St. Paul, MN, USA). These materials represent several categories of GICs commonly used in clinical practice, including conventional glass ionomer cements, resin-modified glass ionomer cements, high-viscosity formulations, and a zinc-reinforced glass ionomer cement. The selection of materials allowed comparison of restorative systems with different compositions and setting mechanisms that may influence their interaction with aqueous environments and their resistance to discoloration.

A total of 25 discs were made from each of the materials used. Each disc was made by placing a split stainless-steel mold (10.5 mm in diameter and 1.5 mm thick) on top of a glass slide with a mylar strip on it, then injecting the material into the mold. After injection, another Mylar strip and glass slide were placed on top of the mold and gentle finger pressure was exerted to allow excess material to be removed.

For the light cured materials, each sample was light cured according to the manufacturers’ instruction using an LED light cure (Woodpecker Medical Instrument Co., Guilin, China). Irradiance was measured at 1000 mW/cm^2^ prior to sample preparation using a digital radiometer (Bluephase Meter II, Ivoclar, Amherst, NY, USA). Self cure materials were allowed to set as per manufacturers’ instructions. Encapsulated materials were activated and mixed according to the manufacturers’ instructions prior to placement in the mold. No additional finishing or polishing procedures were performed after any specimen fabrication. All materials were used in shade A2. Table 1 summarizes the materials used in the study.

### 2.1. Staining Procedure

Five different solutions were used to immerse the samples:

1. Coffee: the solution was made by simmering 15 g of ground coffee (Kurukahveci, Mehmet Efendi, Istanbul, Turkey) one liter of boiling water for 3 min.
2. Tea: the solution was made by steeping 15 g of loose tea leaves (Al-Kbous black tea, Al Kbous Co., Amman, Jordan) in one liter of just boiled water for 5 min.
3. Berry juice: the solution was made by mixing 200 mL of berry juice (Vimto, England, UK) with 1 L of chilled water.
4. Coca-Cola (Coca-Cola, Atlanta, GA, USA) was used undiluted.
5. Distilled water was used as a control.

After the solutions were made up, they were dispensed into wells. Each specimen was stored individually in its respective solution. Based on previously published research, each solution group had 5 samples (*n* = 5) [10]. All the samples were then stored in an incubator at 37 °C (Memmert, Schwabach, Germany). All of the solutions were changed weekly to maintain consistent staining conditions.

### 2.2. Color Change Determination

Baseline (T0) shades were recorded for all specimens using a spectrophotometer (CE7000A, X-rite, Grand Rapids, MI, USA). Prior to each measurement session, the spectrophotometer was calibrated using the manufacturer-supplied white reference tile. To measure the color parameters, each specimen was placed flush against a black background on the holding bracket of the spectrophotometer. The device then measured an area of 8 × 3 mm. The Commission Internationale d’Eclairage (CIE L*a*b*) system parameters were then acquired and used to calculate the ∆E for each time point based on color changes compared to baseline measurements. In the CIE L*A*b* system, L* refers to brightness, a* to the red–green axis, and b* to the yellow–blue axis. The formula used to calculate ∆E is:

$$∆E\;=\;\sqrt{{(L\;\mathrm{post}-L\;\mathrm{baseline})}^{2}\;+\;{(a\;\mathrm{post}-\;a\;\mathrm{baseline})}^{2}\;+\;{(b\;\mathrm{post}-b\;\mathrm{baseline})}^{2}}\;∆E=\sqrt{\;∆\;L2+∆\;a2+∆\;b2}$$

The baseline parameters were recorded at T0, and the post parameters were recorded after T1 (2 weeks of immersion), T2 (4 weeks of immersion), T3 (6 weeks of immersion), and T4 (8 weeks of immersion). ∆E values were obtained at each time point from T1 onwards. At each time point, specimens were rinsed with distilled water and gently blotted dry prior to measurement. The ∆E values were averaged at each time point to give the mean ∆E values for each group.

### 2.3. Water Sorption and Water Solubility

A digital caliper (Mitutoyo 500-197-30) was used to measure the thickness and diameter of each specimen. Immediately after fabrication, specimens were stored under controlled conditions and desiccated to a constant mass before the initial weighing of each specimen (m_1_). A constant mass was when the mass change was less than 0.1 mg between consecutive measurements taken at 24 h intervals. The mass of each specimen was recorded using a digital analytical balance (Scientech^®^—ZSA 210).

Specimens were then immersed in their respective solutions at 37 °C for 8 weeks, after which they were removed, gently blotted dry, and weighed (m_2_). Following immersion, specimens were reconditioned in a desiccator containing silica gel until constant mass was again achieved (m_3_).

The volume (*V*) of each sample was then calculated using the cylinder volume equation: *V* = πr^2^h.

Water sorption and solubility were calculated according to ISO 4049 standards [11] using the following equations:

$$\mathrm{Sorption}\;=\;\frac{m2-m3}{V}\;\mathrm{Solubility}=\frac{m1-m3}{V}$$

where *m*_1_ represents the initial mass after desiccation to a constant mass, *m*_2_ is the mass after immersion and *m*_3_ is the mass after reconditioning to a constant mass. *V* is the volume.

### 2.4. Statistical Analysis

ΔE*ab values were analyzed using a mixed-effects repeated-measures model with time (2, 4, 6, and 8 weeks) as the within-subject factor and material and solution as between-subject factors. Specimen ID was included as a random effect to account for within-specimen correlation across time. Pairwise comparisons of estimated marginal means were performed using Fisher’s least significant difference (LSD) test (α = 0.05).

Water sorption and solubility were calculated from the recorded masses as per the equations shown previously. The results were analyzed separately using two-way ANOVA with material and solution as fixed factors followed by Fisher’s LSD post hoc test (α = 0.05).

All the statistical analysis was done using SPSS Ver. 17 (IBM Inc., Armonk, NY, USA) statistical software at a 0.05 significance level. The assumptions of repeated-measure ANOVA were evaluated. Mauchly’s test was used to assess sphericity and, when violated, Greenhouse–Gessier corrections were applied.

## 3. Results

### 3.1. Color Change (∆E)

**Figure 1:**
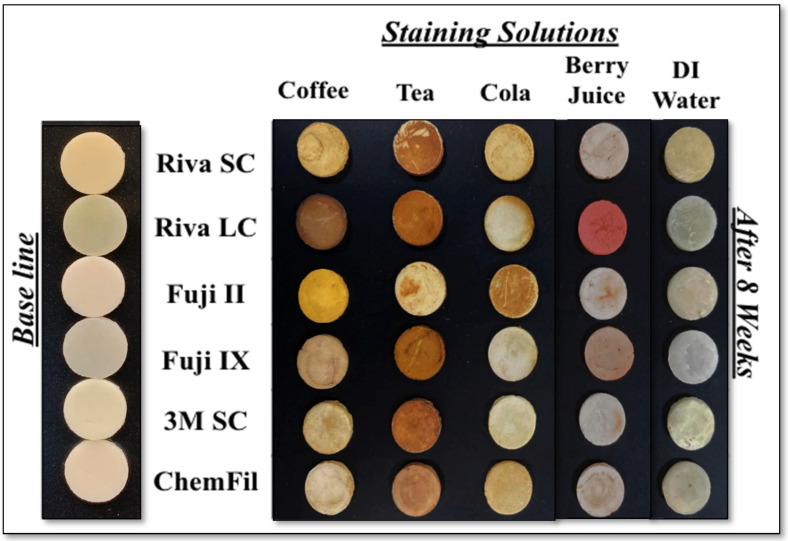
Representative specimens from each group at baseline and after 8 weeks of immersion.

Visual changes were seen in all the tested specimens. However, discoloration in the distilled water group was less pronounced than in chromogenic beverage groups such as tea and coffee. This is illustrated in Figure 1 and is consistent with the spectrophotometer readings.

Baseline spectrophotometer readings are shown in Table 2.

**Figure 2:**
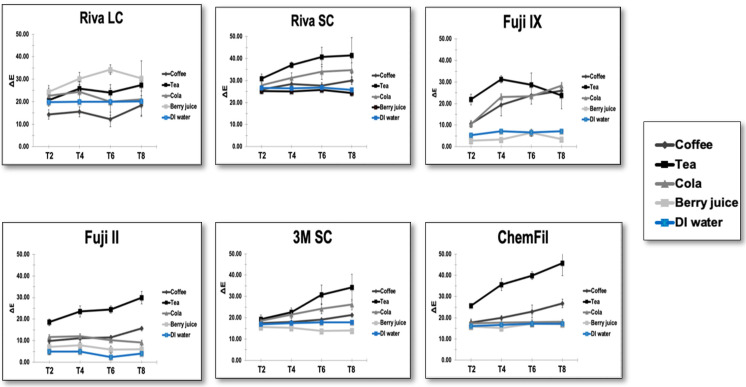
Line graphs showing ∆E values for the six tested materials after immersion in different solutions for 2 (T1), 4 (T2), 6 (T3), and 8 (T4) weeks.

The results of the mixed model repeated-measure ANOVA showed that time, material, and solutions all had a statistically significant (*p* < 0.001) effect on ∆E, with large effect sizes (η^2^_p_ = 0.52, 0.89, and 0.87, respectively). Mauchly’s test indicated that the assumption of sphericity was violated (*p* < 0.001), thus Greenhouse–Geisser corrections were applied. The values of ΔE increased over time from 17.39 (95% CI: 17.07–17.72) at 2 weeks to a value of 22.16 (95% CI: 21.54–22.79) at 8 weeks. The mixed-effects repeated-measures analysis also demonstrated significant interaction effects between material type, immersion solution, and time (*p* < 0.05), indicating that the magnitude of color change depended on the specific combination of restorative material and staining solution over the observation period. Tea produced the highest ΔE values in most materials. Figure 2 illustrates these results.

All the solutions varied statistically from each other regarding ∆E values, except for distilled water and berry juice, which were statistically non-significant. Immersion time determined how much of an effect was seen in the samples, with ΔE values increasing significantly after 4 weeks and peaking at 8 weeks for all groups (*p* < 0.001, repeated-measures ANOVA).

Pairwise multiple comparisons of the material effect showed that ChemFil displayed significantly higher mean ∆E values when compared to other materials (*p* < 0.001) with the exception of Riva LC. Fuji II was the least affected by staining solutions. All the materials’ ∆E values were statistically different from each other (*p* < 0.001) with the exception of Riva LC and ChemFil. The time effect was significant starting at T2 and the influence increased at T3 and T4. It is worth noting the changes in each parameter (L, a, b), along with ∆E values to have a more complete analysis of the color change in the different solutions and materials. This is shown in Table 3.

### 3.2. Water Sorption and Water Solubility

Table 4 summarizes the results of the water sorption and solubility values for all tested materials. Water sorption varied significantly depending on both material composition and immersion solution. Resin-modified glass-ionomer materials generally demonstrated higher water sorption values compared with conventional glass-ionomer cements.

Solubility values were markedly influenced by the immersion medium, with acidic solutions, particularly berry juice, producing the highest solubility values across all materials. Distilled water resulted in the lowest solubility values. Pearson correlation analysis revealed no significant relationship between ∆E and water sorption (r = 0.10, *p* > 0.05) or water solubility (r = 0.18, *p* > 0.05)

## 4. Discussion

Color stability is a critical factor in achieving esthetic success in any restorative material, as it plays a key role in patient satisfaction [12]. Several factors influence color stability; one of which is the coloring agents found in the diet. Ideally, restorative materials should match the patient’s tooth shade and preserve that color semblance over time [12]. In this study, several different GI materials were used to examine the effects of various commonly consumed beverages.

The GI materials chosen for this study are commonly used in dental practices and have studies regarding their performance [8,12]. Shade A2 was chosen due to the fact that it is one of the most common shades of human teeth, thus widely used in clinical practice [7,13]. The choice of beverages is based on those most commonly consumed by the local patient population [7,14,15]. Distilled water was utilized as a control as it has no dietary chromogens [16]. A spectrophotometer was used to measure the color parameters in an objective manner and allow the calculation of differences in color (∆E) obtained using color parameters in the CIELAB color system [7]. Using this method allows minute changes in color to be measured. However, only ∆E values equal to or larger than 3.7 are considered visually detectable [17]. Another study reported that the clinically acceptable color change threshold value was determined as ∆E ≤ 3.3 [18]. In this work, the majority of the ∆E are well above both thresholds as shown in Table 2.

Tested solutions induced various degrees of discoloration after exposure. These findings were consistent with previous work which evaluated glass ionomers’ staining susceptibility to beverages [7,15]. It was clear that immersion time had the main effect on color stability. As immersion time increased, discoloration became more intense. Thus, the null hypothesis was rejected as beverage type, immersion time, and material composition significantly influenced color stability. All of the GI materials tested exceeded the clinically acceptable color changes (ΔE thresholds > 3.3) after 8 weeks of immersion in the five different test solutions. ChemFil had higher ∆E values than others, which made its performance inferior to other GI materials in terms of color stability. Regarding the solutions, immersion in tea produced the highest changes in the ∆E values, leading to darker specimens in nearly all the GIs tested except for Riva LC., which showed the highest ∆E values after immersion in berry juice solution. This result is in agreement with previous work which found that tea was a highly staining solution [10,19]. This may be attributed to the high concentration of tannins and polyphenolic chromogens present in tea compared to coffee. These polyphenolic chromogens and tannins are known to strongly interact with restorative materials and promote pigment adsorption. Thus, coffee showed less staining than tea. Berry juice produced lower color changes compared to coffee and tea, although material-dependent variations were observed. Despite the visual changes noted, there was no significant difference in ∆E values between berry juice and distilled water. This may be due to the high water content of berry juice. Studies have shown that most materials are susceptible to staining by darkly colored beverages, while distilled water causes no perceptible color change [3]. As illustrated in Figure 1 and Table 2, each solution affected the CIE L*a*b* parameters differently. It is worthwhile to note the changes in each parameter (L*, a*, b*), along with ∆E values to have a more complete analysis of the color change in the different solutions and materials. The solution which was the most staining in this work is tea. It caused the most severe discoloration across all materials, with notable reductions in lightness (∆L) and prominent increases in redness (∆a) and yellowing (∆b). Cola was the next ranked in terms of staining. It mainly reduced lightness and increased yellowness across the materials tested. Coffee caused moderate staining, primarily due to reductions in lightness and moderate yellowing. The least staining medium was berry juice even though it increased redness in several materials.

Regarding water sorption and water solubility, the protocol that was used in this study is commonly used in the literature [20]. Water sorption was influenced by the immersion solution and materials. This behavior may be related to differences in material composition, particularly the presence of hydrophilic resin components such as HEMA in resin-modified formulations, which can increase water uptake. Berry juice increased water sorption in Fuji II and Fuji IX but produced the lowest sorption values in Riva SC, Riva LC, ChemFil, and Ketac Fil Plus. These findings indicate that the acidic environment did not increase water uptake. Instead, the sorption appeared to be more affected by the interaction between solution chemistry and material composition, including the stability of the ionic matrix and the presence of resin components. Similar material-dependent responses to acidic storage media have been reported previously [15,20].

Interestingly, water solubility appeared to be more consistently influenced by the immersion medium. Berry juice produced the highest solubility values across all materials, with the most pronounced effect observed in ChemFil. This finding may be explained by the low pH and organic acid content of berry juice which may promote ion leaching and matrix degradation. ChemFil has a zinc reinforced matrix. Previous work has shown that zinc-containing GICs show an enhanced ion release under acidic conditions which may explain the higher solubility found [21]. The influence of the immersion media may also be related to their acidity, as previous studies have reported that commonly consumed beverages such as coffee and tea are mildly acidic, whereas cola and fruit-based beverages exhibit lower pH values due to their organic and phosphoric acid content, which can enhance staining and promote material degradation [15,20]. Coffee and distilled water showed the lowest solubility depending on material while tea and cola resulted in moderate solubility. These findings are consistent with previous studies demonstrating increased solubility of GICs in acidic environments due to breakdown of the acid–base matrix and release of ions [20,22,23].

Statistical analysis revealed no direct correlation between the color change and water sorption or solubility. This finding suggests that staining susceptibility and water related degradation are distinct mechanisms. Previous studies found similar conclusions when examining resin based and glass ionomer materials [9,15]. They found that color stability was more influenced by surface interactions and pigment affinity rather than water uptake on its own.

These findings highlight a potential trade-off between water-mediated maturation, which supports the bioactive behavior of glass-ionomer materials, and increased susceptibility to optical and structural changes during prolonged aqueous exposure. Therefore, while glass ionomer materials exhibit biomimetic advantages through their interaction with the oral environment, their long-term esthetic stability must also be considered when selecting restorative materials.

The clinical implications of these findings are significant. While color stability is a critical consideration for restorations placed in esthetic areas, water sorption and solubility may affect the long-term structural integrity and durability of glass ionomer restorations in all areas. Therefore, material selection should account for both esthetic performance and resistance to water-related degradation, particularly in patients with high consumption of acidic or staining beverages.

As with all in vitro studies, this investigation has limitations. Some of these limitations are that the experimental conditions do not fully replicate the oral environment, where salivary interaction, thermal cycling, mechanical loading, tooth brushing, and intermittent exposure to beverages may influence material behavior. Additionally, the use of standardized disc specimens does not reflect the complex geometries of clinical restorations. Future studies incorporating dynamic oral conditions, longer aging periods, and surface characterization techniques would provide further insight into the long-term clinical performance of GICs.

## 5. Conclusions

All tested GICs demonstrated statistically and clinically significant color changes after 8 weeks of immersion in commonly consumed beverages. Tea exhibited the greatest staining potential across most materials, while berry juice produced the least discoloration. The zinc-reinforced GIC (ChemFil) showed the highest susceptibility to beverage-induced color change, whereas the resin modified GIC (Fuji II) was the least affected by staining solutions.

Water sorption and water solubility were material- and solution-dependent and did not correlate directly with color changes. Fuji II and Fuji IX exhibited higher water sorption values, while berry juice produced the highest solubility values across all materials.

Material composition plays a key role in determining staining susceptibility, water sorption, and solubility. The immersion medium also influences these properties, especially solubility. Clinically, these findings are relevant for both anterior and posterior restorations due to the fact that color stability and resistance to material degradation contribute to the long-term performance of all dental restorations. These results emphasize the importance of considering both patients’ dietary habits and material-specific properties when selecting restorative materials for esthetic areas, as well as managing patient expectations regarding long-term color stability and material durability. They may also assist clinicians in material selection and patient dietary counselling. These findings will help guide the clinician during material selection in esthetic zones, inform patient dietary counselling, as well as manage patient expectations.

## Figures and Tables

**Table 1:** Summary of GI materials used in this study as mentioned by the manufacturers.

Material Name (Abbreviation)	GIC Type	GIC Category	Composition	Manufacturer
Riva Self Cure High Viscosity (Riva SC)	Conventional	High Viscosity	Fluoro aluminosilicate glass, acrylic acid homopolymer, tartaric acid	SDI, Bayswater, Victoria, Australia
Riva Light Cure High Viscosity (Riva LC)	Resin Modified	High Viscosity	Acrylic acid homopolymer, 2-hydroxyethyl methacrylate, glass powder, dimethacrylate cross-linker, acid monomer, tartaric acid	SDI, Bayswater, Victoria, Australia
Fuji IX (Fuji IX)	Conventional	High Viscosity	Aluminosilicate glass, polyacrylic acid powder, polybasic carboxylic acid	GC, Tokyo, Japan
Fuji II (Fuji II)	Resin Modified	Resin Modified	Fluorosilicate glass; HEMA, Al, tartaric acid; polyacrylic acid, water	GC, Tokyo, Japan
ChemFil^®^ Rock (ChemFil)	Zinc Reinforced	Zinc Reinforced	Calcium–aluminum–zinc–fluoro-phosphorus–silicate glass, water, polycarboxylic acid, barium-aluminum-borosilicate glass, tartaric acid, iron oxide pigments, titanium dioxide	Dentsply Sirona, Long Island, NY, USA
3M Self Cure Ketac™ Fil Plus Aplicap™ (3M SC)	Conventional	Conventional	Oxide glass, water, copolymer of acrylic acid—maleic acid, tartaric acid	3M ESPE, St Paul, MN, USA

**Table 2:** Mean and standard deviation of baseline (T0) CIE L*a*b* values for the tested materials.

Material	L	a	b
**Riva SC**	86.77 ± 1.03	0.93 ± 0.13	19.83 ± 0.53
**Riva LC**	66.66 ± 5.33	−1.26 ± 0.46	6.82 ± 1.47
**Fuji IX**	75.67 ± 0.60	0.62 ± 0.15	7.90 ± 0.33
**Fuji II**	59.82 ± 2.20	0.17 ± 0.11	1.93 ± 0.65
**ChemFil^®^ Rock**	89.37 ± 0.79	0.73 ± 0.20	14.35 ± 0.35
**3M Ketac™ Fil Plus (SC)**	82.21 ± 1.29	0.03 ± 0.26	8.18 ± 1.22

**Table 3:** Means and standard deviations of CIE L*a*b* parameters for the materials tested after four weeks of immersion. ∆L: changes in lightness and darkness, ∆a: changes in the red–green axis, ∆b: changes in the blue–yellow axis, ∆E: overall shade change.

Material	Solution	∆L	∆a	∆b	∆E
Riva SC	Coffee	−29.98 ± 1.74	1.26 ± 0.60	0.39 ± 1.33	30.04 ± 1.77
	Tea	−40.55 ± 7.73	7.13 ± 3.61	1.76 ± 3.27	41.39 ± 8.07
	Cola	−34.37 ± 3.17	3.57 ± 1.45	1.26 ± 2.43	34.66 ± 3.35
	Berry Juice	−23.96 ± 1.57	0.57 ± 0.81	−4.18 ± 1.42	24.38 ± 1.43
	DI Water	−25.01 ± 1.38	0.01 ± 0.14	−5.85 ± 1.38	25.73 ± 1.03
Riva LC	Coffee	−17.24 ± 4.56	2.69 ± 0.61	5.31 ± 1.02	18.33 ± 4.27
	Tea	−23.3 ± 3.10	7.58 ± 0.60	11.96 ± 1.42	27.35 ± 2.56
	Cola	−20.61 ± 7.40	1.87 ± 0.92	1.72 ± 3.13	20.96 ± 7.47
	Berry Juice	−24.62 ± 4.18	17.04 ± 7.62	1.70 ± 3.03	30.33 ± 7.72
	DI Water	−20.12 ± 3.40	0.08 ± 0.32	−1.54 ± 1.11	20.21 ± 3.47
Fuji IX	Coffee	−13.86 ± 1.26	2.25 ± 0.75	21.87 ± 1.75	26.00 ± 2.13
	Tea	−16.88 ± 3.70	5.48 ± 2.56	15.49 ± 5.02	23.68 ± 6.20
	Cola	−22.30 ± 5.65	6.89 ± 2.12	15.91 ± 2.66	28.31 ± 6.33
	Berry Juice	−3.13 ± 0.20	−0.17 ± 0.10	1.67 ± 0.09	3.56 ± 0.18
	DI Water	−7.01 ± 0.56	−0.43 ± 0.23	−0.78 ± 0.78	7.11 ± 0.51
Fuji II	Coffee	−12.63 ± 1.76	1.05 ± 0.46	8.94 ± 1.35	15.64 ± 0.73
	Tea	−24.25 ± 3.33	5.25 ± 1.32	16.58 ± 1.21	29.92 ± 2.98
	Cola	−8.74 ± 3.91	0.20 ± 0.44	1.35 ± 1.57	9.18 ± 3.27
	Berry Juice	−5.41 ± 2.75	−0.98 ± 0.27	1.49 ± 0.93	5.95 ± 2.22
	DI Water	−3.85 ± 2.37	−0.88 ± 0.16	−0.49 ± 0.36	4.03 ± 2.32
ChemFil ^®^ Rock	Coffee	−25.60 ± 1.74	2.48 ± 0.59	6.92 ± 2.36	26.71 ± 2.02
	Tea	−40.67 ± 4.33	10.92 ± 2.24	17.53 ± 4.69	45.72 ± 5.80
	Cola	−18.04 ± 0.95	0.52 ± 0.41	0.52 ± 1.59	18.11 ± 1.03
	Berry Juice	−16.93 ± 0.96	0.45 ± 0.48	−0.58 ± 1.13	16.99 ± 0.98
	DI Water	−17.00 ± 0.62	−0.29 ± 0.07	−2.06 ± 0.15	17.13 ± 0.63
3M SC	Coffee	−21.21 ± 0.45	0.36 ± 0.29	1.43 ± 1.42	21.30 ± 0.48
	Tea	−32.09 ± 5.37	6.06 ± 2.65	9.91 ± 3.12	34.20 ± 6.32
	Cola	−24.19 ± 2.09	2.91 ± 0.77	9.3 ± 2.05	26.14 ± 2.41
	Berry Juice	−13.78 ± 2.55	0.42 ± 1.05	1.05 ± 2.5	13.98 ± 2.96
	DI Water	−17.83 ± 1.08	−0.21 ± 0.09	−1.03 ± 0.22	17.86 ± 1.10

**Table 4:** Means and standard deviation of water sorption and solubility for all the tested materials after immersion for 8 weeks in the five solutions.

Material	Solution	Water Sorption (µg/mm^3^)	Water Solubility (µg/mm^3^)
Riva SC	Coffee	97.2 ± 6.4	73.6 ± 5.8
	Tea	98.7 ± 5.1	85.1 ± 6.2
	Cola	92.4 ± 6.9	89.3 ± 5.7
	Berry juice	36.8 ± 8.4	310.7 ± 18.9
	DI water	94.6 ± 6.1	74.9 ± 5.4
Riva LC	Coffee	90.1 ± 5.8	62.7 ± 4.9
	Tea	93.4 ± 6.3	78.6 ± 6.1
	Cola	87.6 ± 7.4	89.2 ± 6.6
	Berry juice	41.2 ± 9.1	266.9 ± 22.4
	DI water	89.3 ± 6.0	62.1 ± 5.2
Fuji IX	Coffee	115.3 ± 6.7	37.9 ± 4.1
	Tea	118.6 ± 5.9	40.2 ± 4.8
	Cola	109.7 ± 6.4	46.8 ± 5.3
	Berry juice	151.9 ± 7.8	401.3 ± 21.6
	DI water	116.8 ± 6.1	41.5 ± 4.6
Fuji II	Coffee	161.2 ± 7.4	72.9 ± 6.2
	Tea	166.8 ± 6.8	83.6 ± 6.9
	Cola	154.7 ± 7.1	92.4 ± 7.3
	Berry juice	189.6 ± 8.3	243.7 ± 18.5
	DI water	158.4 ± 6.6	73.1 ± 6.0
ChemFil^®^ Rock	Coffee	90.6 ± 6.2	94.3 ± 5.8
	Tea	88.9 ± 5.6	101.4 ± 6.3
	Cola	93.7 ± 6.9	109.8 ± 6.6
	Berry juice	58.3 ± 9.7	515.6 ± 31.2
	DI water	91.5 ± 6.1	97.2 ± 5.9
3M Ketac™ Fil Plus (SC)	Coffee	108.1 ± 6.2	69.4 ± 5.1
	Tea	104.6 ± 7.1	78.2 ± 6.4
	Cola	111.3 ± 6.5	92.7 ± 7.2
	Berry juice	71.9 ± 5.3	268.4 ± 19.6
	DI water	98.7 ± 6.8	33.0 ± 5.6

## Data Availability

Data are available from the corresponding author upon reasonable request.
